# Efficacy of four different irrigation techniques combined with 60°C 3% sodium hypochlorite and 17% EDTA in smear layer removal

**DOI:** 10.1186/1472-6831-14-114

**Published:** 2014-09-08

**Authors:** Xiangjun Guo, Hui Miao, Lei Li, Shasha Zhang, Dongyan Zhou, Yan Lu, Ligeng Wu

**Affiliations:** From the Department of Endodontics, School of Stomatology, Tianjin Medical University, #12 Qi Xiang Tai Road, He Ping District, Tianjin, 300070 PR China; Stomatological Hospital of Nankai University, Stomatological Hospital of Nankai University, Tianjin, China; The Department of Stomatology, The second Hospital of Jiaxing, Zhejiang, China

**Keywords:** EDTA, EndoActivator, Irrigant activation, Ultrasonic irrigation, Smear layer, Sodium hypochlorite

## Abstract

**Background:**

Efforts to improve the efficacy of smear layer removal by applying irrigant activation at the final irrigation or by elevating the temperature of the irrigant have been reported. However, the combination of such activation protocols with 60°C 3% sodium hypochlorite (NaOCl) has seldom been mentioned. The aim of this study was to compare the efficacy in smear layer removal of four different irrigation techniques combined with 60°C 3% NaOCl and 17% EDTA.

**Methods:**

Fifty single-rooted teeth were randomly divided into five groups (n = 10) according to the irrigant agitation protocols used during chemomechanical preparation(Dentsply Maillefer, Ballaigues, Switzerland): a side-vented needle group, a ultrasonic irrigation (UI) group, a NaviTip FX group, an EndoActivator group, and a control group (no agitation). After each instrumentation, the root canals were irrigated with 1 mL of 3% NaOCl at 60°C for 1 minute, and after the whole instrumentation, the root canals were rinsed with 1 mL of 17% EDTA for 1 minute. Both NaOCl and EDTA were activated with one of the five irrigation protocols. The efficacy of smear layer removal was scored at the apical, middle and coronal thirds. The Data were statistically analyzed using SAS version 9.2 for Windows (rank sum test for a randomised block design and ANOVA).

**Results:**

No significant differences among the NaviTip FX group, EndoActivator group and control groups, and each of these groups showed a lower score than that of UI group (*P <* 0.05). Within each group, all three thirds were ranked in the following order: coronal > middle > apical (*P* < 0.05). In the coronal third, the NaviTip FX group was better than UI group. In the middle and apical third, the differences were not significant among any of the groups.

**Conclusions:**

Even without any activation, the combination of 60°C 3% NaOCl and 17% EDTA could remove the smear layer effectively, similar to NaviTip FX or EndoActivator, and these three protocols were more effective than UI. However, regardless of different types of irrigation technique applied, complete removal of the smear layer was not achieved, particularly in the apical third.

## Background

Root canal therapy cleans the root canal system through mechanical instrumentation and root canal irrigation [1]. However, during the process of instrumentation, large amount of dentin debris mix with vital and necrotic remnants of pulp tissue, in combination with microorganisms and microbial toxins adhered to the root canal wall, form a smear layer [2]. This smear layer prevents medicaments from penetrating into the dentinal tubules and killing the bacteria within. In addition, the smear layer also decreases the adaptability of filling materials and their penetration into the canal walls, thus reducing their sealing ability [2, 3].

The combined application of sodium hypochlorite (NaOCl) and ethylenediaminetetraacetic acid (EDTA) has been recommended to remove both the organic and inorganic components of the smear layer effectively [2, 4–6]. NaOCl is widely used in endodontic irrigation because of its antimicrobial activity and tissue-dissolving capability [7–9], which removes the organic component of the smear layer. In addition, EDTA, a decalcifying agent, removes the inorganic component of the smear layer [4, 10–12]. Most studies have demonstrated effective but incomplete, smear layer removal with these agents, especially in the apical third of the root canal [13–16]. Higher temperature has also shown to enhance the efficacy of sodium hypochlorite [8, 17–19]. However, even the combination of 60°C 3% NaOCl and 17% EDTA was shown to be inadequate for complete clearance of the smear layer in one study [5].

Various activation techniques such as ultrasonic and sonic systems have thus been introduced in an attempt to enhance the effectiveness of irrigants on smear layer removal. However, it is not clear whether these are effective as inconsistent to previous findings.

The use of Ultrasonic irrigation (UI) contributes to the removal of smear layer [20]. Guerisoli et al. [21] reported the effective removal of smear layers throughout the canal under ultrasonic agitation using NaOCl combined with EDTA plus Cetavlon. However, a conflicting study reported that ultrasonic-activated 5.25% NaOCl and 17% EDTA did not decrease smear layer scores [22].

The NaviTip FX is a brush-covered irrigation needle. A previous report indicated that the efficacy of the NaviTip FX in removing the smear layer in curved root canals, and reported that the use of NaviTip FX with 5.25% NaOCl and 17% EDTA augmented by FileEze (a 19% water-soluble viscous EDTA solution) was the most effective cleaning protocol [23].

EndoActivator is a sonically driven canal irrigation system in which flexible polymer tips do not incise the canal wall [24]. Agitation of the irrigants repeatedly results in significantly more smear layer removal than no agitation (NaviTip), and EndoActivator was found to be significantly more effective than ultrasonic agitation or the CanalBrush in the coronal region of curved root canals [25]. However, EndoActivator was also found not to enhance the removal of the smear layer when compared with Max-i-Probe irrigation with 2.5% NaOCl and 17% EDTA [24].

In an attempt to obtain some clarity and verify or disprove the findings from the studies mentioned above, we applied a higher temperature irrigant and activated it throughout the root canal preparation. The purpose and intent of this study was to compare the efficacy of four different techniques for smear layer removal, including the side-vented needle, UI, NaviTip FX, and EndoActivator protocols, combined with 60°C 3% NaOCl and 17% EDTA.

## Methods

### Specimen preparation

The study was approved by the institutional ethics committee of Tianjin Medical University. Fifty freshly extracted, single-rooted, non-carious maxillary anterior teeth with the patients’ consents were collected and stored in 0.1% thymol solution after the removal of the calculus and periodontal ligaments. The teeth were decoronated to standardize the root length at 15 mm. ISO #10 K-files (Dentsply Tulsa, Tulsa, OK, USA) were inserted into the root canal until they were just visible at the apical foramina at 4× magnification under a surgical microscope (Möller-Wedel International, Wedel, Germany). The working length (WL) was established by deducting 1 mm from this point.

The teeth were randomly divided into four experimental groups and one control group (n = 10). The root canals were prepared with the crown-down technique using ProTaper nickel-titanium rotary instrument (Dentsply Maillefer, Ballaigues, Switzerland) up to size F3.

### Irrigation protocols

#### Side-vented needle group (n = 10)

During the root canal preparation, the root canals were initially irrigated with 1 mL 60°C 3% NaOCl (Septodont, Saint-Maur, France) for 1 min after each instrumentation, and then with 5 mL sterile distilled water followed by 1 mL 17% EDTA (META, Chungbuk, ROK) as the final rinse after the whole instrumentation process. The solutions were delivered by a side-vented needle (24/0.4) (SuYun, Jiangsu, China) placed as deep in the root canal as possible without encountering resistance but not deeper than the predetermined WL minus 1 mm. Finally, the canals were flushed with 5 mL sterile distilled water and dried with sterile paper points.

#### UI group (n = 10)

During the root canal preparation, the root canals were initially irrigated with 1 mL 60°C 3% NaOCl for 1 min after each instrumentation, and then with 5 mL sterile distilled water followed by 1 mL 17% EDTA as the final rinse after the whole instrumentation process. These solutions were delivered by a side-vented needle (24/0.4) and were activated simultaneously with a #15 file (Satelec, Acteon, France) driven by an ultrasonic device (MTS piezo electric unit; Multi Task Cart, Obtura Spartan, USA) at 4/10-scale power in accordance with the manufacturer’s instructions. The working tips used for UI were placed as deep in the root canals as possible without encountering resistance, but not deeper than the predetermined WL minus 1 mm. They were then moved up and down smoothly to facilitate unobstructed backflow of the irrigation solution. Finally, the canals were flushed with 5 mL sterile distilled water and dried with sterile paper points.

#### NaviTip FX group (n = 10)

The procedures in this group were the same as those for the side-vented needle group, but the side-vented needle was replaced with a 30-gauge NaviTip FX (Ultradent Products Inc., South Jordan, UT), which was moved up and down smoothly to facilitate unobstructed backflow of the irrigation solution.

#### EndoActivator group (n = 10)

The procedures in this group were the same as those for the UI group, but UI was replaced with the EndoActivator system (Dentsply Tulsa Dental Specialties). A yellow tip (15/02) was used initially, but was replaced with a red tip (25/04) when a ProTaper F1 instrument was used both vibrated at a frequency of 10,000 cycles per minute (cpm).

#### Control group (no agitation, n = 10)

Using a side-vented needle placed just at the orifice of the root canal, the irrigants (NaOCl and EDTA) were flushed into each canal and left in place for 1 min per canal.

### Scanning electron microscopy

Sterilized cotton pellets were placed in the root canal orifices, and longitudinal grooves parallel to the buccolingual direction were prepared in each specimen using a silicon carbide disk at low speed without penetrating the canal. The teeth were split into two halves along the grooves using an osteotome, fixed in 2.5% glutaraldehyde for 24 h, and then dehydrated in an ascending ethanol series (70%, 80%, and 90%, 15 min per step) before being sputter-coated with gold. The samples were then examined under a thermal field emission scanning electron microscope at the apical third (3 mm from the apex), middle third (7 mm from the apex), and coronal third (11 mm from the apex) in a double-blind test. Photographs were obtained from three randomly selected locations at each site at a magnification of 1000×. Eighteen photographs were obtained from each specimen, giving a total of 900.

Smear layer removal was scored according to the following criteria: (1) the smear layer was completely absent; most of the tubules were patent and debris-free (coronal and middle thirds) or were occluded with sclerotic casts (apical third); (2) the smear layer covered ***<***25% of the canal wall and dentinal tubules; (3) the smear layer was evident in 25%–50% of the canal surface and tubules; (4) the smear layer was evident in 50%–75% of the canal surface and tubules; and (5) the smear layer covered 75%–100% of the canal surface and tubules [26].

Four examiners blinded to the group assignment scored the smear layer removal using the 900 pictures. To eliminate bias, the first 100 pictures were scored more times to ensure intraexaminer consistency and the kappa test results showed good interexaminer agreement, with values > 0.6 for the various categories. Statistical analyses were performed using the rank sum test for a randomised block design and, after rank transformation, ANOVA for a randomised block design. All were performed using SAS version 9.2 for Windows.

## Results

**Figure 1:**
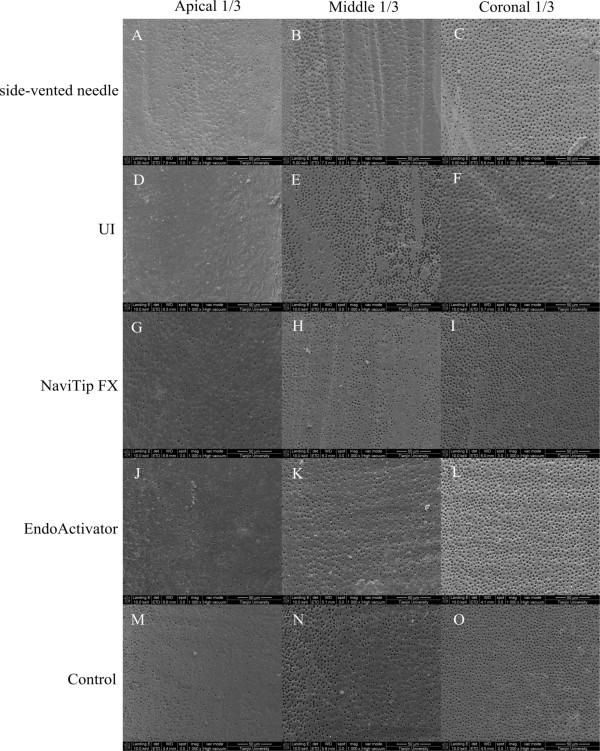
**The smear layer removal of root canal walls of apical, middle, and coronal thirds in five different irrigation techniques displayed in columns (from left); original magnification, 1000×.** Side-vented needle group **(** ***A-C*** **)**, UI group **(** ***D-F*** **)**, NaviTip FX group **(** ***G-I*** **)**, EndoActivator group **(** ***J-L*** **)**, Control group **(** ***M-O*** **)**.

Findings from the comparison of the efficacy of the five different irrigation protocols for smear layer removal are summarized in Figure 1 and Table 1. Statistically significant differences were observed among the five groups (*P <* 0.05). The NaviTip FX, EndoActivator, and control techniques were found to have similar efficacy, and all had lower scores than UI (*P <* 0.05). The UI and side-needle techniques also had similar efficacy since no statistically significant differences were observed.

**Table 1:** **Means and SD values and results of comparison between smear layer scores for different irrigation techniques**

	Side-vented needle		UI		NaviTip		Endo-		Control		
					FX		Activator				
Segment	Mean	SD	Mean	SD	Mean	SD	Mean	SD	Mean	SD	P value
Apical	4.66^a,b^	0.60	4.33^a,b^	0.47	4.66 ^a,b^	1.02	4.42^a,b^	0.78	4.34^a,b^	0.94	> 0.05
Middle	2.83^a,b^	0.71	2.67^a,b^	0.86	2.66^a,b^	0.99	2.00 ^a,b^	1.30	2.34^a,b^	0.56	> 0.05
Coronal	1.48^a,b^	0.35	2.00^b^	0.53	1.58^a,b^	0.79	1.67 ^a^	1.03	1.58 ^a,b^	0.61	< 0.05
Total	2.83^a,b^	1.30	3.08^a^	0.29	2.58^b^	0.98	2.16^b^	1.38	2.42^b^	1.24	< 0.05

Means with the same superscripts are not statistically significant (*P* > 0.05).

Regarding comparisons of the canal thirds (Figure 1), statistically significant differences were observed among the coronal, middle, and apical thirds (*P <* 0.05). Efficacy was greatest in the coronal third followed by the middle third and then the apical third in all groups.

Statistical analysis of the efficacy of the different irrigant agitation protocols in each canal third (Figure 1, Table 1) revealed that the EndoActivator technique was more effective than UI in the coronal third (*P <* 0.05), but no other statistically significant differences were observed. All of the techniques appeared to have similar efficacy in the middle and apical thirds (*P* > 0.05).

## Discussion

According to the literature, irrigant activation protocols that are only applied at the final irrigation can improve the efficacy of smear layer removal [27, 28]. However, there are few data available regarding the application of agitation protocols throughout the preparation. Further, the combination of such protocols with an initial rinse with 60°C 3% NaOCl has seldom been mentioned. In our study, the smear layer was removed using 60°C 3% NaOCl and 17% EDTA in combination with five different protocols including the side-vented needle, UI, NaviTip FX, and EndoActivator techniques, as well as no agitation throughout the preparation.

We found that even without any agitation, the combination of 60°C 3% NaOCl and 17% EDTA was effective at removing the smear layer, and had a similar efficacy to that of the NaviTip FX and EndoActivator techniques. Thus, the non-activated and activated irrigation techniques had similar efficacy, which contradicts the conclusions of Caron et al. [27]. The difference might be due to the efficacy of NaOCl in the removal of the organic component. This is primarily dependent on the activated chlorine concentration, which is increased to 6%–9% when 5% NaOCl is heated to 60–85°C for 4 h [29]. All of the NaOCl used in this study was heated to 60°C, which ensured an adequate activated chlorine concentration for removing the smear layer. In addition, the volume of the irrigant might have influenced smear layer removal as with no agitation, merely leaving the irrigant within the root canal for 1 min allowed for a greater volume of irrigant inside. At the same time, the space taken up by the activation appliance (NaviTip FX or EndoActivator) could have made a difference since the root canal is such a tiny space; the appliance could have reduced the volume of irrigant within the root canal and thus decreased its efficacy. This discrepancy might have been compensated for by the scrubbing action and mechanical activation, which could explain the finding of similar efficacy.

Guerisoli [21] and Kuah [30] reported that ultrasonic agitation could effectively remove the smear layer. However, in the present study, the NaviTip FX, EndoActivator, and no agitation were more effective at removing the smear layer than UI. This finding is most likely to be partly associated with the fact that the NaviTip FX is an irrigation needle that is covered with bristles that enhance smear layer removal during scrubbing [31]. In addition, the EndoActivator has a polymer-based tip with a smooth surface, so it does not cut the root dentin [24]; hence, no new smear layer is formed. In addition, the diameter of the EndoActivator tips increased with successive enlargement of the root canals. Switching from yellow to red tips enabled deeper insertion and closer adaptation to the dimensions of the shaped canals. Use of the highest vibrational frequency of 10,000 cpm might also have ensured better smear layer removal. As for UI, it was required that the ultrasonic tips should not contact the root canal wall during the irrigation process. However, it was difficult to keep the tips from touching the walls of the root canal [27], which most likely resulted in incisions in the canal walls triggering the formation of a new, undesirable smear layer and decreasing the efficacy of smear layer removal. Finally, no agitation was more effective for smear layer removal than UI, which might be due to the larger volume of irrigant used and the fact that no new smear layer was formed as no canal wall incisions were made.

The efficacy of the irrigation protocols in terms of smear layer removal in the coronal, middle, and apical thirds ranged from excellent to poor, probably owing to the following reasons: (1) As the diameter of the root canal gradually decreased from the coronal to apical third, the volume of the irrigant decreased, which decreased the liquid backflow. Thus, less irrigant was flushed into the apical third than the middle and coronal thirds; (2) fewer dentinal tubules were found in the apical third compared with the middle and coronal thirds, and the extent of mineralisation increases with age [5].

## Conclusions

Even without any activation, the combination of 60°C 3% NaOCl used as irrigant during root canal preparation and 17% EDTA as the final irrigant was effective for smear layer removal and showed similar efficacy to the NaviTip FX and EndoActivator techniques; all three were more effective than UI. However, regardless of the type of irrigation technique applied, complete removal of the smear layer was not achieved, particularly in the apical third.
